# Beyond dichotomous thinking: a process perspective on diabetic foot disease

**DOI:** 10.1080/2000625X.2017.1380477

**Published:** 2017-09-28

**Authors:** Gustav Jarl, Lars-Olov Lundqvist

**Affiliations:** ^a^ Department of Prosthetics and Orthotics, Faculty of Medicine and Health, Örebro University, Örebro, Sweden; ^b^ University Health Care Research Centre, Faculty of Medicine and Health, Örebro University, Örebro, Sweden

**Keywords:** Diabetic foot, patient compliance, shoes, orthotic devices, foot ulcer, diabetes complications, diabetes neuropathies, diabetes mellitus

## Abstract

**Background**: Diabetic foot (DF) disease causes severe suffering around the world, and appropriate self-care activities are needed to prevent and treat this condition. However, all too often, self-care activities are less than optimal and clinicians find themselves unable to influence them in a positive direction. Clinicians’ and researchers’ mental models of the DF tend to be dichotomous: either the patient has or does not have an active ulcer or other DF disease. This mode of thinking hides the long-term perspective of DF disease, where patients’ previous experiences and expectations for the future influence their current behavior. Thus, there is a need for a different perspective on DF disease to better understand patients’ perspectives and thereby improve self-care, leading to more effective prevention and treatment.

**Objective**: To present a novel framework, the process perspective on the DF, which can explain inadequate self-care behaviors not easily understood with a dichotomous perspective, and how they can be changed.

**Results**: Three fictive clinical examples are used to illustrate how the process perspective on the DF can be used to understand how patients’ previous experiences and expectations for the future influence their current behavior. In particular, this process perspective is used to understand how patients’ beliefs and behaviors are sometimes self-reinforcing, resulting in stable behavior patterns, here referred to as ‘DF cycles’. These cycles are quite common in clinical practice but are difficult to analyze using a dichotomous perspective on DF disease. The process perspective on the DF is used to analyze specific ‘vicious’ DF cycles of inadequate patient behavior and to find ways to transform them into ‘virtuous’ DF cycles, resulting in effective prevention and treatment.

**Conclusions**: The process perspective on the DF seems suitable for understanding inadequate patient behaviors not easily understood with a dichotomous perspective on DF disease, opening up new avenues for clinical practice and research to help patients live a life with long remission phases, few relapses, and a high quality of life.

## Introduction

Some 415 million people in the world have diabetes, and it is estimated that, annually, 9.1–26.1 million of them will develop a foot ulcer [1,2]. These foot ulcers and associated amputations reduce quality of life (QoL) of patients and their families and put a substantial economic burden on society [3,4]. Medical interventions are essential, but effective prevention and treatment also demand that patients conduct appropriate self-care activities, such as wearing appropriate footwear, inspecting the foot daily, and lubricating dry skin [5]. However, adherence to diabetic foot (DF) self-care activities is often less than optimal [6], and clinicians might feel frustrated when finding themselves unable to understand the reasons for the low adherence and uncertain about how adherence can be improved. In this study, we hypothesize that the problems of understanding and changing inadequate DF self-care is related to our mental models of DF disease.

It is well known that patients’ mental models of DF disease, including their experiences and interpretations of causes and mechanisms, influence their behaviors and what actions they take [7–9]. For example, patients who have had a minor amputation or think that wearing therapeutic shoes is important to prevent complications report higher adherence to wearing therapeutic shoes [10,11], while patients who think that they cannot prevent foot ulcers report lower preventive foot self-care [12]. Similarly, clinicians’ mental models of DF disease might impact what actions they choose to take, but this is seldom discussed in the literature. Clinical thinking in the DF field sometimes resembles a dichotomy: either the patient has or does not have an active foot ulcer. This dichotomous thinking is even more evident in research, where each study usually focuses on either prevention or treatment of DF disease, although a few studies do include both perspectives [13]. Guidelines also tend to fall into this way of thinking, where recommendations are frequently categorized into recommendations for prevention and recommendations for treatment [5,14]. Naturally, any perspective highlights certain aspects of reality and downplays others. The dichotomous perspective suggests that different actions need to be taken during the prevention and treatment phases. Consequently, the dichotomous perspective downplays the long-term perspective of DF disease, which might include several phases of prevention and treatment, in which patients’ previous experiences and expectations for the future influence their current behavior. Thus, to better understand patients’ inadequate self-care behaviors and how they can be improved, we need a mode of thinking that acknowledges that DF disease is a process over time. This process is the focus of a novel framework, the process perspective on the DF, which is proposed here.

First we briefly review behavior theories that have been applied to DF self-care. Second, we describe the main themes of the process perspective on the DF. Third, we use three clinical examples to illustrate how the process perspective can be used to understand and change certain patterns of patient behaviors not easily understood or changed using the dichotomous perspective on the DF. Finally, we discuss potential implications of the process perspective on the DF for clinical practice and research.

### Behavior theories and DF self-care

The behavioral sciences provide a large number of theories and models that seek to explain how behaviors can be changed. Many of these theories and models have been studied in the context of general diabetes self-care [15], but their relevance for DF self-care has been investigated to a much lesser extent.

The health belief model [16] describes the likelihood of engaging in health-promoting behavior as a function of perceived threat of the health condition (perceived seriousness and susceptibility), perceived benefits and barriers to engaging in the behavior, and cues to action. In the 1980s, self-efficacy was added to the health belief model, denoting beliefs about one’s capability to perform a certain behavior [17]. Three studies of DF self-care practices found that one or more components of the health belief model were evident [18–20]. However, another study [21] found no such correlations. Hjelm et al. [12,22–24] used the health belief model as part of their theoretical framework in four qualitative studies on DF self-care in relation to gender and national background, also including the locus of control concept in their analyses. Locus of control denotes whether an individual perceives the outcome of an event as a result of one’s own behavior (controllable) or to some external forces beyond one’s control (uncontrollable), such as others, mere chance, or fate [25]. In one study, DF self-care correlated positively with internal locus of control and negatively with a chance form of external locus of control [26]. Another model that has been proposed to understand DF self-care behavior is the common-sense model of illness behavior [7,27]. It describes how patients give meaning to their diagnosis and symptoms out of previous lay knowledge, perceived significant others, and personal experiences, and from this construct a mental model and make decisions on what actions to take [28]. One study found that common-sense misperceptions predicted inferior DF self-care and a correct understanding of the causal mechanisms of ulceration enhanced self-care [29]. Other theories and models, such as the information–motivation–behavior skills model and the transtheoretical model, have been proposed to facilitate changes in DF self-care behaviors [30], but empirical research on their effectiveness to improve DF self-care is still lacking.

In summary, research on how to change DF self-care behaviors is still in its infancy. Some concepts and models seem promising for future investigations but their value and explanatory power might be unnecessarily limited if solely used from a dichotomous perspective on DF disease. For instance, studies in the future might find that differences in terms of the common-sense model, health belief model, locus of control, or self-efficacy explain some of the variance in self-care behavior during prevention and treatment phases. Still, this approach does not take into account the recursive nature and bi-directional influence of the mental model and actions or experiences, resulting in both the model and behavior changing over time as phases of prevention and treatment vary. Thus, a framework beyond the dichotomous perspective on the DF is needed to take the bi-directional influence and long-term perspective into account in clinical practice and research.

## New framework: the process perspective on the DF

**Figure 1.**
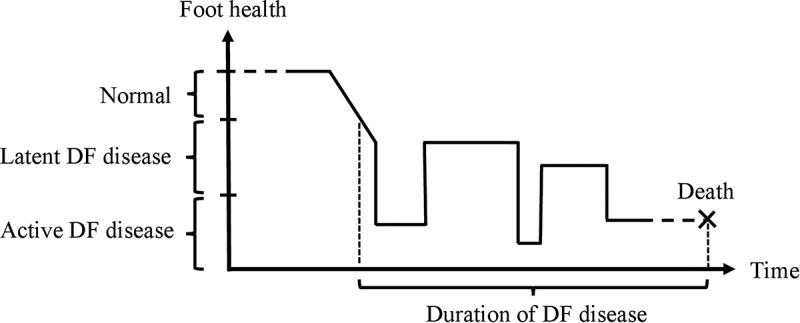
Visualization of the process perspective on the diabetic foot (DF). The patient’s foot health starts at a normal level but at some point in life starts to deteriorate and foot health is categorized as latent DF disease. For the rest of the patient’s life, he or she remains in this category with possible outbreaks of active DF disease.

Before the framework of the process perspective on the DF is described, a few words on the terminology may be appropriate. The International Working Group on the Diabetic Foot defines the DF as ‘infection, ulceration or destruction of tissues of the foot associated with neuropathy and/or peripheral artery disease in the lower extremity of people with diabetes’ [31]. In the process perspective on the DF, this is referred to as ‘active DF disease’. Furthermore, the term ‘latent DF disease’ is used in the process perspective on the DF to denote phases of increased risk of active DF disease, including both the remission phases between the phases of active disease [32] and the period of increased risk before the first onset of active disease. The central principle of the process perspective on the DF is that DF disease is not considered to be a dichotomy, but rather a process over time. Thus, the patient is not viewed as belonging to one of two categories, prevention or treatment, but viewed as standing in the midst of a process, with a history of experiences and expectations for the future, all relevant to the patient’s mental model of DF disease and current self-care behavior. Prevention and treatment are thus not two categories to put patients in, but different phases of the same process. Figure 1 illustrates the basic view of DF disease according to the process perspective on the DF.

The patient’s foot health starts at a normal level but at some point in time starts to deteriorate, for example, with the onset of peripheral neuropathy or peripheral arterial disease, and reaches the threshold for a diagnosis of latent DF disease, classified as the first stage of DF disease. The latent DF disease category is characterized by increased risk of active DF disease and can be further stratified into different risk categories depending on what risk factors are present [33]. In the course of DF disease there are alternating phases of active DF disease and latent DF disease (at risk or in remission). Importantly, the patient is never cured of DF disease; foot health does not return to normal. The remission phases are characterized by high risk of new onset of active DF disease, and the patient has latent or active DF disease until death. Thus, the first diagnosis of latent DF disease marks the onset of a lifelong commitment to preventing outbreaks of active DF disease. In this sense, DF disease resembles other lifelong diseases and disabilities, where the patient needs to learn to live with the condition. The ultimate aim is to help patients to live a life with long remission phases (latent DF disease), few and short relapses (active DF disease), and a high QoL. To achieve such an aim, both medical interventions and lifestyle changes are usually needed, and compromises are often necessary. However, we cannot achieve effective prevention and treatment if we do not understand the beliefs and behaviors of the patients, sometimes manifesting in behavior patterns that keep repeating over time, no matter whether they are constructive or not.

### Virtuous and vicious DF cycles

Here, the term ‘DF cycle’ is coined and is used to refer to a set of patient beliefs and behaviors that are self-reinforcing through a feedback loop and produce a distinct behavior pattern. The DF cycle is considered *virtuous* if it results in effective prevention and treatment, and *vicious* if it results in ineffective prevention and treatment. Below we use three fictive examples to illustrate how the process perspective on the DF can be used to analyze and understand vicious DF cycles: why they develop, what maintains them, and how they can be transformed into virtuous DF cycles. The examples focus on adherence to wearing offloading devices, but the conceptual framework of process perspective is applicable to other aspects of DF self-care as well.

### Clinical example 1: low adherence during remission and repeated reulcerations

**Figure 2.**
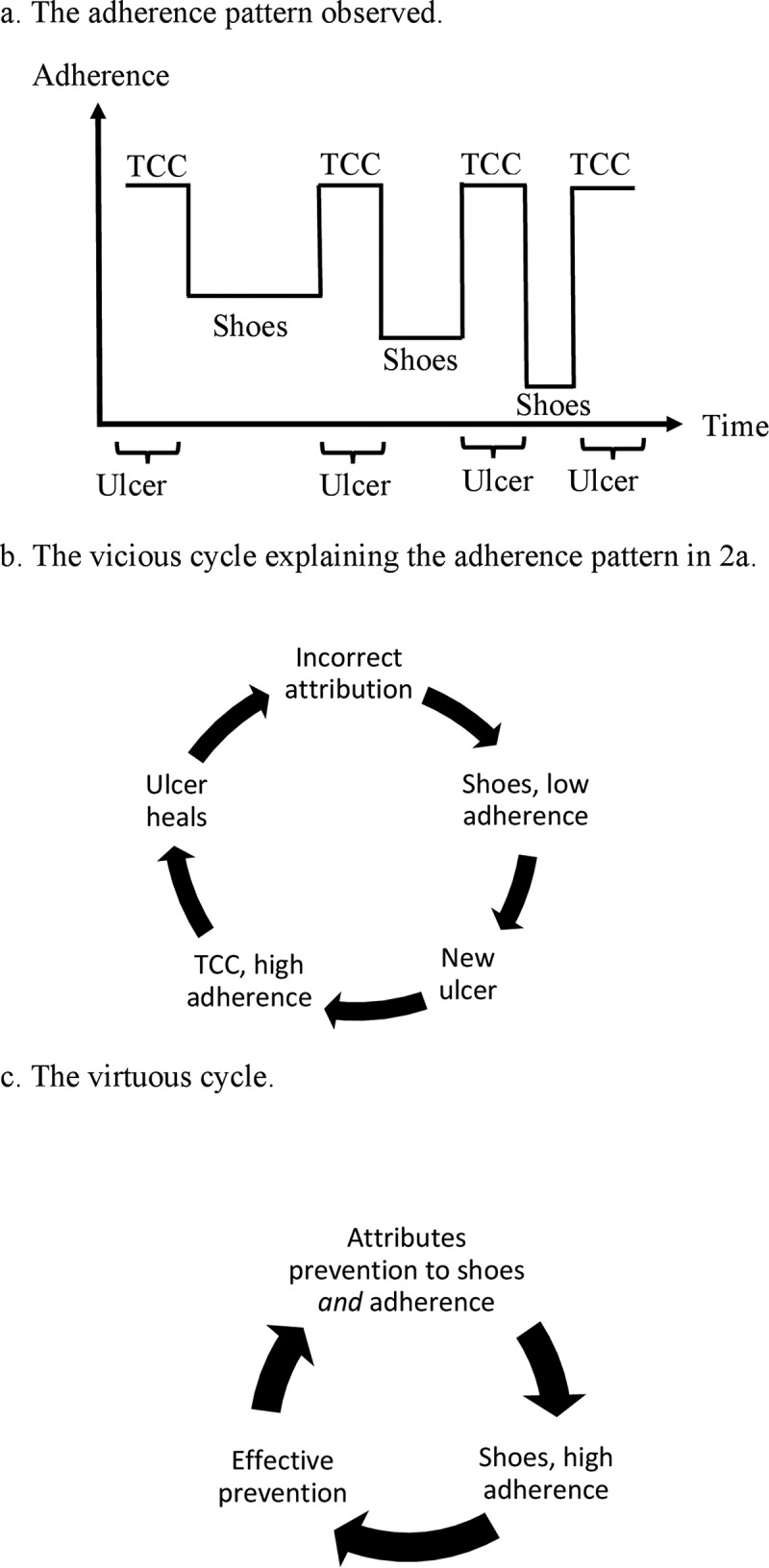
A patient is treated with a total contact cast (TCC) during the ulcer phases, but shows low adherence to wearing therapeutic shoes during remission (a). This can be explained by a vicious cycle (b) of too much emphasis on the type of devices and too little emphasis on adherence, leading to low adherence to wearing therapeutic shoes with reulcerations as a consequence. If the patient’s adherence to wearing therapeutic shoes can be increased long enough for him to see healing progress the vicious cycle can be transformed into a virtuous cycle of high adherence (c).

David has had a metatarsal head ulcer in several periods for the past four years. He has been treated with a total contact cast (TCC) during the treatment phases and with therapeutic shoes during remission phases. However, each time the ulcer heals, it recurs within a few months after healing. In addition, the remission phases get shorter and shorter over time (Figure 2(a)). David declares, ‘The shoes are no good! When I wear a cast the ulcer heals, but as soon as I change to shoes it comes back!’

From a dichotomous perspective on DF disease, David’s case is problematic. Guidelines often categorize prevention and treatment as separate issues, and David is treated according to the guidelines. These guidelines recommend using a non-removable device such as TCC to offload and heal plantar foot ulcers [14,34] because many patients show low adherence to wearing removable offloading devices [35–40]; they also recommend that therapeutic shoes are used after healing to prevent reulceration [14,34], as the use of non-removable devices is not feasible during remission.

Using the process perspective on the DF, a different picture of David’s case appears. When analyzing his behaviors and beliefs over time, we realize that following the guidelines can actually cause a vicious DF cycle to develop. Each time David’s ulcer heals, two things are changed simultaneously: the type of device is changed (from TCC to therapeutic shoes) and the adherence to wearing the device is changed (from 100% with TCC to low adherence with shoes; Figure 2(a)). As the change of devices is more obvious to David than the change in adherence level, he attributes the reulcerations to the shoes *per se* rather than to low adherence to wearing them. This reinforces David´s belief that therapeutic shoes cannot prevent ulcers (incorrect mental model of the DF) and consequently further reduces his adherence to wearing them, which becomes a self-fulfilling prophecy confirming his incorrect mental model. Every turn in this vicious cycle (Figure 2(b)) fosters a fatalistic view that ulcers cannot be prevented, a common view among patients [24], despite evidence for the preventive effect of wearing therapeutic shoes [41].

To transform this vicious cycle into a virtuous cycle we need to address David’s incorrect attribution of reulcerations to the shoes. First, we can try to convince him that his therapeutic shoes redistribute pressure effectively and that he just needs to wear them more. Mueller et al. [42] demonstrated how visualizations of pressure measurements can be used to educate patients about the offloading effect of shoes when peripheral neuropathy makes the patient unable to recognize high pressure. Second, we can try to counteract David’s incorrect attribution by using the same type of device during the treatment and remission phases, so that he cannot attribute reulcerations to change of devices. Jarl and Tranberg [43] sealed a therapeutic shoe with a plastic band to render it irremovable during ulcer treatment, and removed the seal when the ulcer had healed. Thus, the patients continued to use the same shoes after ulcer healing. However, whether this approach translates into higher adherence after healing and lower reulceration rates has yet to be investigated. By using the approaches described by Mueller et al. [42] and by Jarl and Tranberg [43], we might be able to increase David’s adherence to wearing therapeutic shoes and thereby reduce his risk of reulcerations, to establish a self-reinforcing virtuous cycle (Figure 2(c)). In time, his high adherence may form a habit without any further need for external reinforcement to maintain it.

### Clinical example 2: diminishing adherence and chronic ulcer

**Figure 3.**
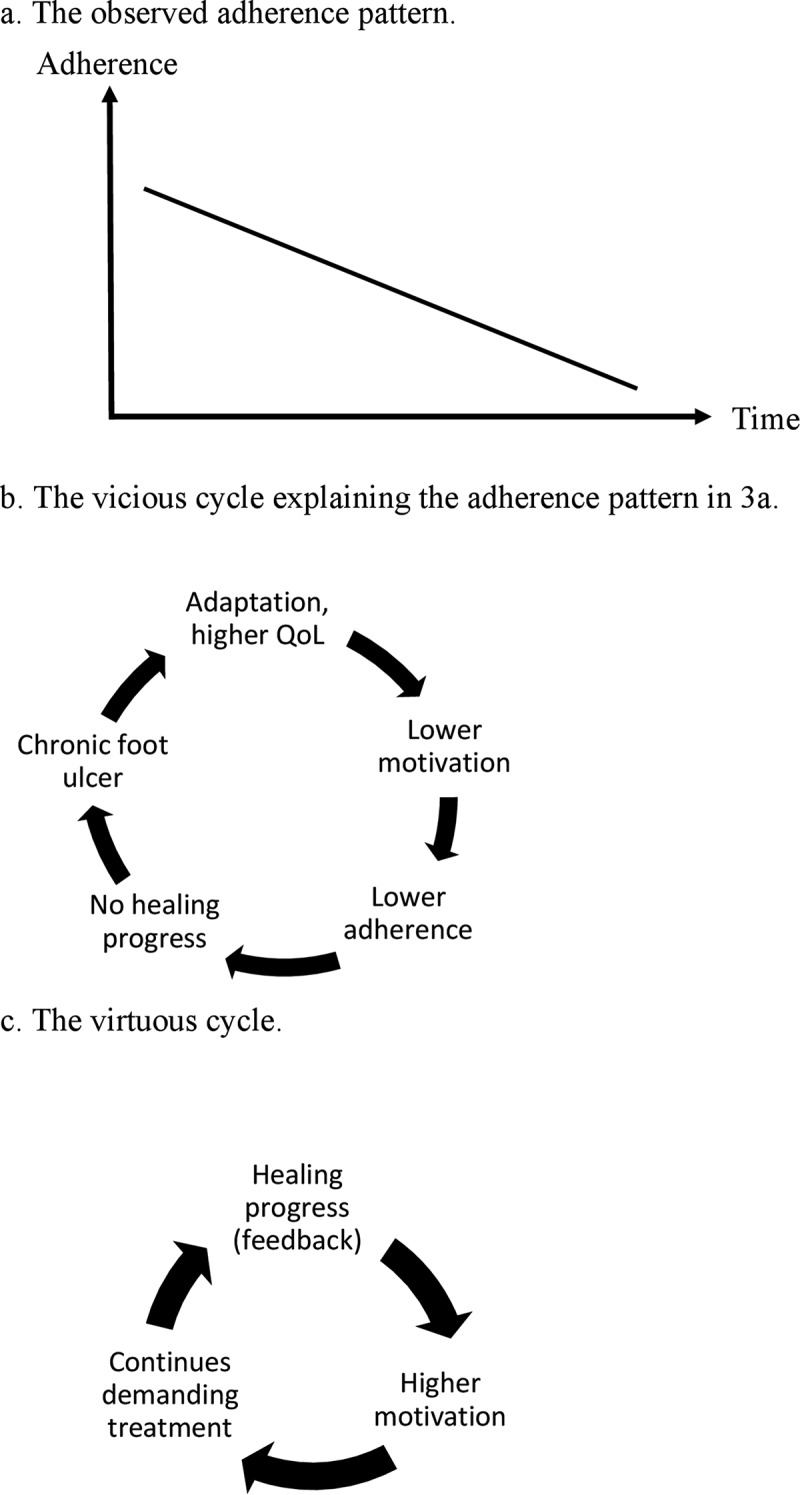
A patient with a chronic ulcer shows decreasing adherence to wearing her therapeutic shoes over time (a). This can be explained by a vicious cycle (b) of adaptation, higher quality of life (QoL), lower motivation, lower adherence, and chronic ulcer. If we can change the relative costs and gains in QoL implied by treatment and healing, thereby increasing her motivation for a more effective treatment regimen, a virtuous cycle may be reached where her motivation is further increased by giving her feedback on the healing progress (c).

Maria has had a toe ulcer for two years without progress towards healing and has been prescribed therapeutic shoes. Initially, she was bothered by the ulcer and wore her therapeutic shoes most of the time. Now she wears her shoes less and less (Figure 3(a)). When clinicians suggest treatment with TCC, she is not interested. Maria responds, ‘Sure, a cast can heal the ulcer, but it is just so inconvenient!’

From a dichotomous and short-term perspective, Maria’s case illustrates a rather frustrating situation: the patient and clinician agree on what treatment would heal the ulcer, but the patient says no because she finds the treatment more burdensome then the symptoms. From the process perspective on the DF, where the perspective is extended backwards and forwards in time, the situation appears somewhat different. The process perspective on the DF focuses on how the patient’s mental model of the DF has been shaped by previous experiences, and what this model tells the patient to expect in the future. DF ulcers often result in lower QoL for the patient [4]. Still, patients who live with chronic health conditions or disabilities can adapt to their situation and report a good or excellent QoL [44]. One way this adaption might operate is by changing expectations to better match the current experiences [45]. On the one hand, as Maria learns to live with her condition there is a better match between her expectations and her experiences, which results in a perceived higher QoL. On the other hand, this may reduce her motivation for treatment because healing of the ulcer will not improve her present QoL substantially. Instead, a TCC would temporarily reduce her QoL because it restricts mobility and makes it difficult to sleep [34]. Thus, Maria feels that the small expected gain in QoL is not worth the temporary cost.

In this vicious cycle (Figure 3(b)) it is important to intervene at an early stage to prevent the cycle from spiraling downward. If no progress is made toward healing, the treatment regimen should be changed to a more effective, and potentially more demanding, regimen before the patient has adapted to the situation and lost motivation. Furthermore, to transform Maria’s vicious cycle into a virtuous cycle (Figure 3(c)) her motivation for treatment needs to be addressed. This can be achieved by modifying the relative QoL costs of treatment and QoL gains of ulcer healing. First, we can increase her motivation if we reduce the negative impact on QoL of the proposed treatment regimen. For example, an instant TCC, i.e., a walker rendered irremovable [46], might be more convenient to Maria than a TCC, as no changes of casts are necessary. Thus, her treatment cost, in terms of QoL, would be lower, thereby increasing her motivation. Second, we may increase her motivation if we can increase the difference in QoL she expects between living with or without a foot ulcer in the future. Although her QoL may be reasonable at present, the ulcer might get infected in the future, causing her health to deteriorate; her mental model might not have taken this into account. Thus, the relative QoL gain of ulcer healing might be higher than her present mental model leads her to expect. If we can explain this to her, her motivation for a more demanding treatment regimen such as TCC might increase. Once the more effective treatment regimen has been initiated, she is further motivated to continue the treatment by receiving feedback on the healing progress in the form of photographs or measurement of ulcer size from the healthcare provider (Figure 3(c)), producing a virtuous DF cycle. As the new treatment regimen becomes part of her daily routine, the need for external feedback diminishes.

### Clinical example 3: intermittent adherence and chronic ulcer

**Figure 4.**
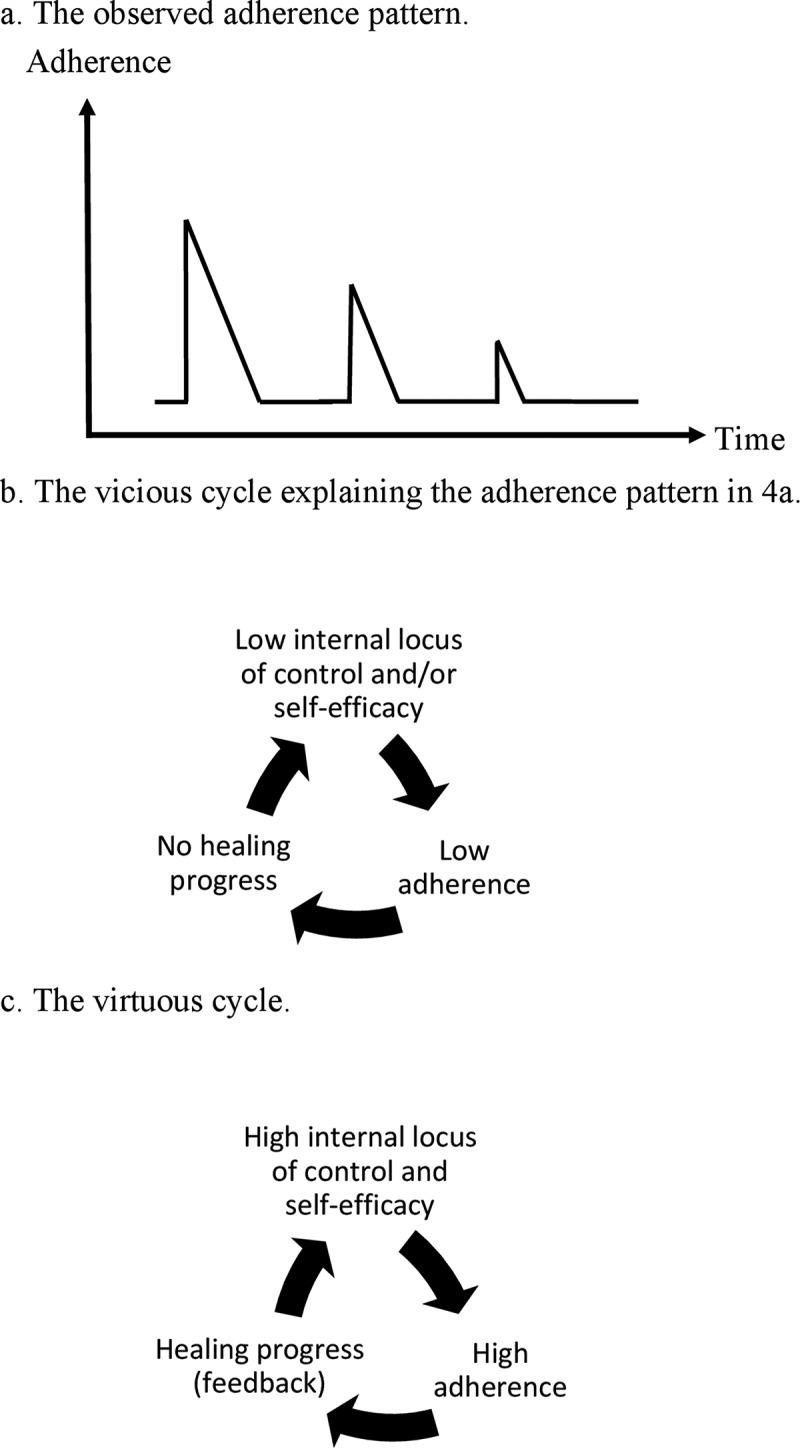
A patient repeatedly tries to improve adherence to wearing his cast walker, but after each visit to the clinic his adherence quickly diminishes (a). This can be explained by a vicious cycle of low adherence, low internal locus of control, low self-efficacy, and unsuccessful treatment (b). If the treatment regimen is tailored to maximize his chances of success, thereby improving his internal locus of control, self-efficacy, and adherence, a virtuous cycle may be reached (c). Feedback on treatment progress is used to further strengthen his adherence.

Joseph has had an ulcer under the heel for three years. He has been prescribed a cast walker but shows low adherence to wearing it. At each appointment he seems highly motivated to improve his adherence, but his intentions fall short each time (Figure 4(a)). Joseph admits, ‘I know that I have to shape up. It just doesn’t fit my lifestyle.’

From a dichotomous and short-term perspective on the DF, this is a scenario where it is tempting to blame the patient for his low self-discipline and adherence. Still, by applying the process perspective on the DF, we extend the perspective into the past and the future to better understand his situation. In this vicious DF cycle, negative experiences from repeated failures with adhering to wearing the walker influence Joseph’s interpretation of the situation and his expectations for the future, further lowering his chances of successful treatment. The first issue is Joseph’s locus of control, that is, to what extent he believes that he has control over the healing of the ulcer (internal locus of control) or attributes this to factors beyond his control (external locus of control) such as fate or his diabetes diagnosis *per se*. The second issue is Joseph’s self-efficacy, that is, his belief in his ability to adhere to wearing the walker whenever he is active. Repeated failures might change his mental model of DF disease and make him believe that adherence does not matter (low internal locus of control) and/or that he is unable to adhere (low self-efficacy). In either case, this becomes a self-fulfilling prophecy with low adherence and lack of treatment progress confirming his incorrect model. To break this vicious cycle (Figure 4(b)) we need to tailor the treatment to increase Joseph’s internal locus of control and self-efficacy, resulting in higher adherence. For example, if the reason for his low adherence to wearing the walker is forgetfulness, a device with audio feedback when the device is not being worn could be useful [47]. If the reason for low adherence is that the walker impairs his balance [35], therapeutic shoes might be more appropriate. The important thing is to tailor the treatment regimen to maximize his chances of being successful and seeing progression of the healing. Through this positive experience, his internal locus of control and self-efficacy will increase, thereby increasing his chances of success in the future, producing a virtuous DF cycle (Figure 4(c)). However, two things are important to consider when creating this virtuous cycle. First, Joseph’s adherence must reach a high enough level for the healing to progress; otherwise he may fall back into his vicious DF cycle. Second, feedback from healthcare providers on treatment progress is crucial: Joseph’s internal locus of control, self-efficacy, and adherence will not improve in response to objective treatment progress, as only subjective treatment progress can change his mental model. Thus, feedback in the form of photographs or measurements of ulcer size should be given to him at each appointment to demonstrate his treatment progress and strengthen his continued adherence until adherence has become part of his daily routine.

## Discussion

This paper presents a novel view of DF disease, the process perspective on the DF. The process perspective highlights the fact that prevention and treatment are two parts of the same process, and that they need to be taken into consideration simultaneously. Some clinical examples were given to illustrate how the process perspective on the DF can aid clinical understanding and guide interventions, potentially more effectively than a dichotomous mode of thinking. As a clinical model, the process perspective on DF disease has some advantages over the dichotomous perspective: it describes DF disease as a chronic condition with latent and active phases that are parts of the same process, which calls for continuity of care, greater patient involvement, and a long-term perspective. Ideally, clinicians should think about, and educate the patient about, how to prevent new ulcers during the treatment phase and how to treat new ulcers (early identification, referral, and intervention) during the remission phase. In other words, clinicians and patients should be proactive and stay one step ahead: they should plan for prevention during treatment and for treatment during prevention.

In clinical practice, the process perspective on the DF suggests three overarching approaches. First, clinicians should stimulate the development of virtuous DF cycles. Second, clinicians should prevent vicious DF cycles from developing by early identification of, and intervention against, inadequate beliefs and self-care behavior. Third, clinicians should break vicious DF cycles by identifying the factors that maintain these patterns of behavior and applying interventions.

In clinical research, the process perspective on the DF could be used to open up new avenues for research, such as how patients’ mental models of DF disease are affected by different interventions and experiences, and thereby have consequences for future self-care behavior. For example, studies could investigate whether adherence to wearing therapeutic shoes after healing differs if shoes or TCC were used to offload the ulcer, or whether a patient in remission who develops an ulcer while wearing therapeutic shoes loses faith in the shoes, resulting in low adherence to wearing them during the treatment phase. Another avenue of research is how to give feedback to patients to maintain or increase motivation for adherence. Often, ulcer healing is a long-term process in which the cost (in terms of QoL) is immediate and certain while the outcome may be distant and uncertain. In these cases, feedback on the healing progress is important to keep patients motivated and engaged in the treatment. In other cases, such as ulcer prevention, the goal is not an improvement but non-deterioration, which might be even more difficult to give feedback on. In these cases, research is needed on whether feedback on the effects of preventive measures can be used to keep patients engaged. For example, quantifying the amount of fissures or callus that builds up between the appointments to the podiatrist, or visualizing plantar pressures with and without therapeutic shoes. In the end, the positive self-care behavior, at first motivated by positive feedback, could stabilize into an established habit, without further need of external reinforcement.

Hopefully, the process perspective on the DF will facilitate novel modes of thinking about DF disease in clinical practice and research. The examples used for illustration in this paper focus on adherence to wearing offloading devices. Still, we believe that the process perspective and DF cycles have a wider applicability in DF clinical practice and research, and we hope that this perspective will stimulate future development in the field. A potential limitation of the study is that the model is based on clinical experience; studies are needed to test the model with empirical data.

## Conclusion

The process perspective on the DF seems suitable to understand patient behaviors not easily understood with a dichotomous perspective on DF disease, opening up new avenues for clinical practice and research to help patients live a life with long remission phases, few relapses, and a high QoL.
